# The Impact of Airline’s Smart Work System on Job Performance of Cabin Crew

**DOI:** 10.3390/ijerph191912414

**Published:** 2022-09-29

**Authors:** Yongjin Jung, Haeok Liz Kim, Sunghyup Sean Hyun

**Affiliations:** 1 School of Tourism, College of Social Sciences, Hanyang University, 222 Wangsimni-ro, Seongdong-gu, Seoul 04763, Korea; 2 Computational Social Science Center, Hanyang University, Seoul 04763, Korea

**Keywords:** efficiency, convenience, service effectiveness, pride, job satisfaction, team performance, organizational commitment, turnover intention

## Abstract

Extant studies in medical and educational fields have demonstrated that employees’ device use (smartphones, tablet PCs, etc.) can enhance job performance. Correspondingly, global airline companies have made substantial investments to enhance passenger services. An earlier study examined the impact of flight attendants’ technology usage on job satisfaction by investigating the causal relationship between the benefits of tablet PC use, job performance, and its consequences. Based on the literature review, four advantages of technology use were derived: (1) efficiency, (2) convenience, (3) service effectiveness, and (4) pride. Additionally, three consequences of job satisfaction were derived: (1) team performance, (2) organizational commitment, and (3) turnover intention. Empirical data were collected from 208 flight attendants working for a South Korean airline, which provided tablet PCs for its employees. Data analysis revealed that work efficiency, convenience, and pride had a significant and positive impact on job satisfaction. However, flight preparation did not show a similar impact. This study is the first to investigate the benefits of using technology in the airline industry. Furthermore, it examined the convergence of airline management and information technology. The findings provide managerial implications for airline companies that are considering providing tablet PCs to flight attendants.

## 1. Introduction

The service industry has made protracted attempts to use technology to enhance efficiency. Undoubtedly, the aviation industry is extensively dependent on information and communication technology (ICT) [1,2,3], not only for optimizing procedures and processes of airline operations management [4,5,6,7], but also for in-flight entertainment and customer services.

In recent years, it has become customary to search and book flights, check-in, and obtain information on departures and arrivals through mobile devices such as smartphones or laptops. An increasing number of airline companies are also providing Wi-Fi on board. Technical innovations have been introduced across the aviation sector [8]. Presently, airline companies are becoming more app-based platform enterprises through collaborations with startups.

Recently, airline companies have also been making attempts to incorporate new technology in order to distinguish themselves from their rivals and survive intense competition. These companies are now providing personal tablets to their employees working at the forefront. This is currently being implemented in approximately 20 airlines worldwide (Delta Airlines, United Airlines, JetBlue Airways, British Airways, KLM, Air France, Lufthansa, Alaska Airlines, Iberia, Emirates Airlines, Etihad Airways, Qantas, ANA, etc.).

Among these, A Airlines (pseudonym used for anonymity) is the first Korean airline to provide tablet PCs to all flight attendants. A airline introduced the “A-tab” system, a smart work platform (a kind of smart work platform that can submit work-related information and reports through a tablet PC), to increase the cabin crew’s work efficiency [9]. Many studies have been conducted on how incorporating personal devices such as smartphones or tablets into jobs achieves improved work performance. However, most of the existing research has focused on medical and educational institutions, and none on flight attendants in the aviation field.

Accordingly, the purpose of this study is to (1) create and test a cognitive positive effect model for the introduction of new technology in which flight attendants use tablet PCs for work; (2) determine the factors that maximize job satisfaction among flight attendants; and (3) investigate the impact of the cognitive positive effect of adopting new technology on team performance/organizational commitment/turnover intention. Consequently, this study will provide theoretical and practical guidance for the adoption of new technology in flight attendants’ work in the airline industry.

## 2. Literature Review

### 2.1. Adaptation of New Technology, Use of Tablet PCs by Flight Attendants

A tablet PC refers to a computer equipped with a flat touch screen that uses a digital pen or finger as the main input device, as opposed to a keyboard or mouse, and the screen is usually 5–14 inches. Tablet PCs are generally Wi-Fi, 3G, or higher wireless internet connection-enabled, and are equipped with a mobile operating system such as Apple’s iOS or Google’s Android. Tablet PCs are convenient to carry, and compared to other media and communication technologies, have been gaining popularity, as consumers’ preference for them seems to be increasing. Therefore, adaptation of work using tablet PCs is increasing in the aviation industry. As mentioned earlier, about 20 airlines worldwide have begun using tablet PCs at work.

Considering that safety is extremely important in the aviation industry, flight attendants are required to carry safety manuals always comprising over 1000 pages when flying. As the *Aviation Act* mandates that safety manuals be immediately revised whenever circumstances change or a new aircraft is introduced, these manuals need to be updated and printed several times a year. However, the dissemination of tablet PCs can reduce the cost of printing and distributing the training manuals, while the latest information can be updated instantly and applied in the field.

Mirvis, Sales, and Hackett (1991) studied the implementation and adoption of new technology in organizations regarding the impact on work, people, and culture. The implementation of new technology may improve work relationships, skill utilization, and job performance. These in turn improve organizational effectiveness and the quality of the work life of employees [10].

In the next sections, we discuss in detail the type of cognitive positive effects that occur when flight attendants adapt to new technology. These can be largely divided into four categories: (1) efficiency, (2) convenience, (3) service effectiveness, and (4) pride.

#### 2.1.1. Efficiency

Many studies have demonstrated that employees’ use of technology-intensive devices for work simplifies work preparation and increases efficiency. Employee efficiency in the workplace may be increased through bring-your-own-device (BYOD) programs [11].

Ghosh et al. defined the benefits of BYOD, which refers to the use of personal mobile devices instead of handouts printed by employees or the necessity to log into websites to prepare for work [12]. BYOD increases employee productivity, as corporate information and organizational data are readily available on personal mobile devices such as smartphones, PCs, and laptops.

Mohsen Hakami conducted a survey of 69 female students from Sharura Science and Arts University at Nazran University in Saudi Arabia to gauge their learning satisfaction through Nearpod, an educational application software, using personal mobile devices [13]. If a teacher uploads lecture materials using Nearpod’s application, the students can study using their personal mobile devices or interact with students and teachers in real time for interactive classes. The study found that the students expressed satisfaction with various multimedia learning materials tailored to individual needs on their mobile devices, in a switch from the traditional teaching method that involves the use of textbooks and paper [13].

Additionally, Singh, Chan, and Zulkefli (2017) found that the use of mobile computing devices among students for higher education provide an opportunity for better performance, productivity, convenience, and a promise of mobility [14].

In addition, as work-related information that should be obtained from various sources can be accessed from a single integrated device, efficiency can be maximized in terms of time [12].

In the case of the aviation industry, prior to the use of tablet PCs, flight attendants prepared a manual for flight attendant safety, accessed an intranet that can only be read within the company, selected the necessary information from various kinds of flight-related information, and wrote it down manually. Subsequently, a group of flight attendants used to participate in the brief. Currently, all the information, including the safety manual, can be contained in a single application, making the task of preparing briefings easy, which increases efficiency [9].

Based on these studies, we proposed the following hypothesis.

**Hypothesis** **1** **(H1).** *Efficiency positively influences job satisfaction.*

#### 2.1.2. Convenience

The second positive cognitive effect obtained by introducing the new technology is convenience. Convenience has not been clearly defined in the literature, and it is appropriate to understand convenience as a multidimensional construct. To examine the convenience of services, Brown proposed a conceptual framework with five dimensions: time, place, acquisition, use, and execution [15]. Based on Brown’s theory, this study defines the dimensions of perceived convenience for flight attendants regarding the use of tablet PCs.

(1) Time dimension: This refers to the degree of perception according to which the use of a tablet PC allows a person to perform work at a convenient time. In most airlines, flight attendants can check flight information only from their company premises. However, using a company-issued tablet PC allows them to verify flight information at a convenient time, even before going to work. However, it should be noted that this dimension does not mean “time saving.”

(2) Place dimension: This refers to the degree of perception according to which work can be performed in a more convenient place using a tablet PC. While flight attendants are on their way to the airport, they can update flight information through their personal mobile devices even while traveling. In addition, if they use Wi-Fi on a flight, they can communicate with the ground staff and send necessary information during flights.

Based on the research, a self-service technology (SST) theory was developed, which implies that consumers can choose their own services without the help of staff [15]. The remaining dimensions are summarized on the premise that cabin attendants can be considered customers in terms of technology use.

(3) Execution speed dimension: This refers to the degree of recognition according to which a tablet PC (or iPad) is convenient to use in the process of performing work [16]. Speed has been selected as an important factor in numerous qualitative studies on SST [17,18,19]. Task processing speed is defined as the time required for users to actively complete a selection through SST [20]. “Perceived convenience” in handling tasks with self-service can have a strong impact on perceived speed. Accordingly, Farquhar and Rowley emphasized that the convenience of a service is related to the concept of execution, a process involving the time taken to perform a task [21].

With the use of tablet PCs, flight attendants feel that the speed of execution of tasks has increased because they can receive the latest information through updates rather than waiting on an aircraft for information delivered by ground staff [14].

(4) Accuracy dimension: This refers to the degree of perception according to which the use of a tablet PC has increased accuracy in the process of one’s performance. Transaction accuracy or processing ability for customer needs is an important factor in evaluating the quality-of-service experience [22,23]. A qualitative study by Wolfinbarger and Gilly showed that customers who had the convenience of starting and stopping online transactions on their own had a higher perception of information accuracy than those who had offline interactions with service employees [24].

According to Airline Trend (2017), the Emirates airline started taking flight meal orders from business class passengers using an MOD (meal ordering devices) system [25]. In the case of in-flight meal service, the awareness of information accuracy among flight attendants is higher when passengers order a meal through a personal monitor than when they take meal orders directly from the passengers. This also makes flight attendants’ work easier. Correspondingly, the convenience of using a tablet PC for flight attendants is expected to increase.

Based on these studies, we proposed the following hypothesis.

**Hypothesis** **2** **(H2).** *Convenience positively influences job satisfaction*.

#### 2.1.3. Service Effectiveness

Information technology (IT) has been widely used in collaboration with various businesses. Among them, group support systems (GSSs) enable communication between participants in a collaborative group, allowing them to interact simultaneously to make decisions and address issues. IT has been increasingly used to support collaborative work in a variety of business contexts. GSSs allow participants in a collaborative group to interact simultaneously and anonymously to generate ideas, make decisions, and resolve issues. GSSs can be very effective when used by groups to perform tasks that do not require information-rich communication, such as planning and creativity tasks involving generating ideas.

Combining Daft and Lengel’s information richness theory with McGrath’s “task circumflex” results in a theory of a “task/technology fit,” which suggests that GSSs-mediated communication may be very effective for certain task types, but less effective or detrimental for other task types [26,27].

Specifically, techniques that provide simple information, such as yes or no, for general tasks that do not require expertise or creative discussion seem effective. Therefore, the use of GSSs, which provide simple information, can have a significant impact on handling tasks efficiently [26,27,28]. Effectiveness and job satisfaction are increased because employees are working with devices of their own choosing and are accordingly more familiar with the technology [9]. The consumerization of information technology (CoIT) suggests that organizations can benefit from the implementation of the BYOD concept and boost employees’ functionality at work [29]. The CoIT is a natural phenomenon, given that mobile devices have become ubiquitous. In terms of flight attendant work, the use of tablet PCs is also helpful because it simplifies ordering in-flight meals or duty-free items.

BYOD has brought significant convenience and advantages to business activities enhancing work flexibility and efficiency [30]. For example, in the healthcare industry, medical staff use IT devices at work. When a doctor or nurse uses a personal mobile device for work purposes, it reduces the time for paper-based documentation so that patient care and treatment will receive enough attention. Therefore, studies have shown that this has a positive effect on employees’ perceived work productivity [31,32,33,34]. In addition, research has suggested that productivity can be improved owing to faster communication and information retrieval [35,36,37,38].

Eslahi, Naseri, Hashim, Tahir, and Saad (2014, April) found that personal mobile devices can be used to promote employees’ satisfaction and work efficacy [30].

Based on these studies, we proposed the following hypothesis.

**Hypothesis** **3** **(H3).** *Service effectiveness positively influences job satisfaction.*

#### 2.1.4. Pride

Psychological ownership refers to the cognitive–affective state in which a consumer experiences a sense of ownership, regardless of actual legal ownership, believing that “This is mine!” about the target. It indicates the relationship between someone “who has been closely related to himself” and someone else [39]. This includes things, ideas, and others. Three distinct paths lead to psychological ownership: (1) dealing with an ownership target, (2) having a close relationship with the target, or (3) putting money or effort into the target [40].

Furthermore, psychological ownership is experienced more strongly when the target activates their identity, enhances their sense of self-efficacy, provides stimulation, or makes them feel familiar, like being at home [39]. Psychological as well as physical possession becomes a part of the expanded self [41], and by giving value to their possessions, they intrinsically have pride [42].

In the IT field, when consumers choose technology artifacts such as applications and communication functions, the psychological ownership of technology can be strengthened. The ownership of technology provides a means to value oneself. Therefore, it motivates one to increase one’s value through pride, which is an evaluation according to which one’s ability is superior to that of others [43].

By comparing oneself with others, one can achieve satisfaction and confidence or experience disappointment and frustration [44]. In addition, developmental psychologists have noticed that for young children, simply owning an object before others is enough to elicit feelings of ownership [44]. Therefore, it is highly likely that the effect will be strengthened in public (private) consumption situations because pride can be self-conscious and social. People who act in public places have stronger pride than those who act in the absence of others, and behaviors in public places can trigger implicit social comparisons [44,45,46]. Therefore, ownership, whether psychological or legal, is essentially a social construct as it has limited meaning in the absence of social comparison.

Accordingly, research results have been developed according to which individuals can vicariously experience pride through the accomplishment of other team members in the group [47,48]. Similarly, Lee and Hyun suggested that flight attendants who feel proud of their organization can have a psychologically positive effect on other members, which also positively influences active service behavior [49].

Among Korean airlines, A Airlines first introduced a tablet PC to the flight attendants’ work. In the airline industry, flight attendants provide in-flight services with tablet PC applications, which helps them gain psychological ownership and increase the pride of flight attendants. We suggest that flight attendants will be proud of the response of passengers and of flight attendants from other airlines.

Based on these studies, we proposed the following hypothesis.

**Hypothesis** **4** **(H4).** *Pride positively influences job satisfaction.*

### 2.2. Job Satisfaction

Job satisfaction refers to a positive emotional state in which the evaluation results in showing how one’s job or job experience is enjoyable or positive [50,51,52,53]. In addition, some scholars define it as a combination of feelings and beliefs that organizational members experience toward their current duties [54,55].

According to Lyons et al., job satisfaction can be effectively enhanced by using implicit correction factors (e.g., personal development, useful technology) rather than explicit encouragement factors (e.g., wages) [56].

Pitichat found that authorizing the use of a smartphone for work can result in the following outcomes: (1) freedom to choose the method of working, (2) deeper relationships formed among co-workers through the use of inner social communications, and (3) a convenient and practical way of sharing information [57]. The results of the study indicated that these factors induce positive job satisfaction, which eventually leads to higher productivity [57].

Jeong et al. conducted a study on 113 hotel employees from seven five-star hotels in U.S. cities (New York, Los Angeles, San Francisco, Miami, Chicago, Philadelphia, and Washington DC). It was recognized that using a mobile device at work (BYOD) is beneficial to overall work performance, as it brings a sense of self-efficacy, which leads to greater satisfaction and positive results in extending the tenure of employees [58].

### 2.3. Team Performance

A team is defined as a group of people working interdependently to achieve a common goal, working together in a trustworthy manner [59]. Kalisch et al. suggested that teamwork consists of four essential components: involving more than two employees together to achieve a shared goal or objective, having clear and established roles within the team, ensuring that each member of the team understands the roles of all members, and working together through collaboration in order to achieve the stated goal [60].

Teamwork is an essential attribute of the aviation industry. Many studies have shown that the service rendered by flight attendants is enhanced through teamwork and the synergy it creates among the staff, rather than individual abilities while working independently [60,61,62,63,64]. Ku, Chen, and Hsu showed that flight attendants with high job satisfaction were found to be more active in dealing with complex and newly updated service manuals [65].

Park conducted a study on 322 flight attendants of K Airlines in Korea and found that the greater the team member’s job satisfaction, the higher the team’s overall job performance [66]. Therefore, to improve team performance, it is imperative to identify the individual tendencies of flight attendant team members, form appropriate teams accordingly, and assign suitable tasks.

Based on these studies, we proposed the following hypothesis.

**Hypothesis** **5** **(H5).** *Job satisfaction positively influences team performance.*

### 2.4. Organizational Commitment

Organizational commitment refers to the degree of unity that individual members of an organization experience toward their organization [67]. This means identifying oneself with a particular organization by being active and positive, and expressing a willingness to contribute significantly to the organization. This leads to commitment and attachment toward a certain organization, leaving oneself with a strong desire to have a long-lasting relationship [67,68].

A study on 275 flight attendants in Taiwan by Ku et al. found that flight attendants with high job satisfaction have an enormous organizational commitment toward their work and are more proactive in responding to complex and newly updated airline service manuals and behavioral bases [65].

Testa (2001) conducted a study to examine the relation between job satisfaction and organizational commitment in the context of service environment. The findings suggest that an increase in job satisfaction increased organization commitment and service effort [69].

Russ and McNeilly (1995) studied the relationship between job satisfaction and organizational commitment using experience, performance, and gender as moderators. The results indicated that performance and experience moderate the relationship between job satisfaction and organizational commitment [70].

Based on these studies, we proposed the following hypothesis.

**Hypothesis** **6** **(H6).** *Job satisfaction positively influences organizational commitment.*

### 2.5. Turnover Intention

Turnover intention refers to the intention of an employee affiliated with a company to leave in the near future after working for a certain period [71]. Considering that flight attendants’ service duties are intensive in nature and involve relatively high labor costs, managing turnover is an important issue for most airlines. Flight attendants, who work as frontline employees, play a critical role in directly engaging with passengers and delivering flight services [72].

Lee and Lee investigated the factors of job satisfaction that may lower turnover intentions [73]. Their study was conducted among 201 Korean flight attendants of an airline by subdividing job satisfaction into work, company, and performance satisfaction. Consequently, the job satisfaction obtained by establishing a friendly relationship with co-workers and introducing and learning new technology greatly reduces the turnover rate [73].

Based on these studies, we proposed the following hypothesis.

**Hypothesis** **7** **(H7).** *Job satisfaction negatively influences turnover intention.*

## 3. Results

### 3.1. Study Design and Participants

In this study, we used a self-report questionnaire survey and convenience sampling to obtain responses from flight attendants of A Airlines.

The inclusion criteria were as follows: (1) flight attendants currently working for the airline, and (2) flight attendants with experience using a tablet PC provided by the airline.

During data collection, all flight attendants who participated in the survey were informed that the collected information would remain confidential and would be destroyed after the analysis was completed. After the participants gave their consent, they were provided with a link to an online survey via social networking sites or email. In addition, face-to-face questionnaires were administered within a flight attendant briefing room in the company or through individual meetings.

**Figure 1:**
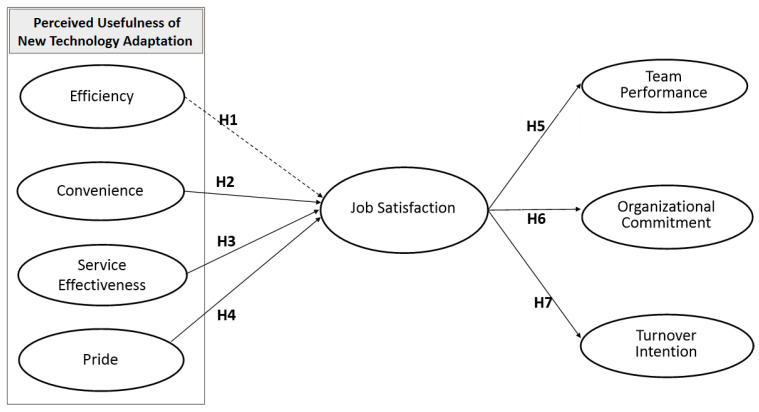
Research Model.

Overall, 215 questionnaires were administered both through online and face-to-face methods between 15 August and 15 September 2020. Of these, seven responses that seemed unreliable were eliminated, while the remaining 208 questionnaires were included in the analysis. Figure 1 presents the research model.

### 3.2. Measures

To empirically measure the nine theoretical concepts proposed in this study, measurement items verified in the existing literature in various fields (flight attendant competency, communication, psychology, etc.) were applied as follows.

Three questions on flight preparation derived from a previous study on the four dimensions of the cognitive positive effect of new technology introduction, Ghosh et al. [11], four questions of convenience derived from Brown [15], Farquhar, and Rowley [21], three questions on efficiency derived from Niehaves et al. [29], and three questions of pride derived from Dommer and Swaminathan [42] and Lee and Hyun [49] were measured using an interval scale.

Job satisfaction was measured using three questions adopted by Kristensen and Nielsen [55] and Lyons et al. [56].

Team performance was evaluated using three questions adopted by Boshoff and Allen [63].

Organizational commitment was measured using three questions adopted by Mowday et al. [67] and Ku et al. [65].

Turnover intention was measured using three questions adopted by Mobley [71] and Chen [72].

After creating the initial questionnaire based on the aforementioned measures, we asked the questionnaire participants to respond to each question on a 5-point Likert scale ranging from “strongly disagree” (1 point) to “strongly agree” (5 points). To ensure the validity of the measures used in this study, we conducted a preliminary interview survey with a focus group consisting of flight attendants of A Airlines before administering the questionnaire. The questionnaire conducted a pilot test with 10 flight attendants of A airline in Korea with more than 3 years of experience prior to the field survey. Seen as A airlines introduced tablet PCs in July 2019, flight attendants with more than 3 years of experience can compare the advantages of tablet PCs. Four core factors were derived through the focus group interview (efficiency, convenience, service effectiveness, pride), but the environmental factors were not suitable for the content of the study, so they were modified and, finally, four cognitive positive factors were derived.

Next, we conducted a pilot test with 30 flight attendants to check the readability of the questionnaire. Based on the preliminary survey, we made several improvements and adjustments, and administered the questionnaire after a final check by a professional group specializing in the subject. We obtained a Cronbach’s α > 0.7, suggesting that the scales used in this study were reliable.

For the development of the measurement tools in this study, four benefits of technology adaptation were derived: (1) efficiency, (2) convenience, (3) service effectiveness, and (4) pride. Additionally, three consequences of job satisfaction were derived: (1) team performance, (2) organizational commitment, and (3) turnover intention.

The equivalent scale used a 5-point Likert scale (1 = “not at all,” 5 = “very much so”), where a value of 1 is the respondent’s strong negative view, and a value of 5 implies the respondent’s strong positive view.

### 3.3. Data Analysis

First, a frequency analysis was performed to understand the general characteristics of the study participants. Second, a confirmatory factor analysis (CFA) was conducted to evaluate the validity of the measurement model, convergent validity and discriminant validity were verified, and Cronbach’s α coefficient was checked to verify the reliability of the measurement tool. Third, a structural equation model analysis (SEM) was conducted to verify the relationship between the variables. For statistical analysis, IBM SPSS 25 and AMOS 25 were used, and statistical significance was determined based on a significance level of 5%.

## 4. Research Results

### 4.1. Demographic Profile of the Respondents

Table 1 shows the respondents’ characteristics. To achieve the objectives of our study, we collected a sample of 208 individuals, of which 24 were male (11.5%) and 184 were female (88.5%). Among these, 41 participants (19.7%) were aged 20–29, 123 (59.1%) were aged 30–39, 39 (18.8%) were aged 40–49, and 5 (2.4%) were aged >50. Our sample consisted of 90 single (43.3%) and 118 married participants (56.7%). During the survey period, 18 participants (8.7%) had graduated from a professional college, 158 (76.0%) from college, 15 (7.2%) were currently enrolled in a graduate school, and 17 (8.2%) had graduated from a graduate school or above.

Additionally, 4 participants (1.9%) had less than 2 years of experience, 25 (12.0%) had 2–5 years of experience, 83 (39.9%) had 6–10 years of experience, 49 (23.6%) had 11–15 years of experience, and 47 (22.6%) had more than 15 years of experience. Regarding the participants’ job titles, 90 participants (43.3%) were stewards/stewardesses, 84 (40.4%) were assistant pursers, 29 (13.9%) were pursers, 3 (1.4%) were senior pursers, and 2 (1.0%) were chief pursers. Based on salary, 5 participants (2.4%) received an annual salary of less than KRW 30 million, 45 (21.6%) received KRW 30–40 million, 83 (39.9%) received KRW 41–50 million, 44 (21.2%) received KRW 51–60 million, and 31 (14.9%) received a salary of more than KRW 60 million per annum.

### 4.2. Results of Confirmatory Factor Analysis and Reliability Analysis

Table 2 presents the results of the CFA. In this study, the CFA of the measurement model was performed to verify the unidimensionality of the latent variables. As a result of the analysis, the chi-square values for the model fit were CMIN = 677.930, DF = 314, and *p* = 0.000, indicating that the model fit was satisfactory (chi-square/df = 2.159). CFI = 0.919, IFI = 0.920, TLI = 0.903, which satisfied the incremental fit index and was judged to be a suitable model [72]. In addition, if the RMSEA value was 0.05 or less (0.05–01 is appropriate), it was judged to be appropriate, but RMSEA = 0.078 was judged as acceptable [73].

#### Convergence Feasibility Verification and Validation

Table 3 presents the results of the conceptual validity analysis. Composite reliability (C.R.) is above 0.7, and the average variance extracted (AVE) value is above 0.5, so an internal consistency verification is not required. The concentration feasibility was secured for this purpose. Discriminant validity verification and latent factor measurements were performed using the mean variance (AVE) value [74].

As shown in Table 3, discriminant validity was secured because the AVE value was larger than the square of the correlation coefficient between the variables.

In the concept validity analysis in Table 4, it was found that there was no multicollinearity because the correlation coefficient of each concept did not exceed 0.8.

### 4.3. Analysis of Structural Models and Hypothesis Validation

#### SEM and Goodness of Fit

In this study, SEM analysis was performed to verify the theoretical hypothesis presented in previous studies. Several factors can affect the changes magnitude in fit statistics, such as sample size, pattern of non-invariance, model complexity, and ratio of sample size [75]. According to the analysis, CMIN = 746.136, DF = 332, *p* = 0.000, indicating that the model fit was appropriate (chi-square/df = 2.247). If the RMSEA value is 0.05 or lower (0.05–01 is appropriate), it is judged to be suitable. RMSEA = 0.078, which was considered acceptable [76]. CFI = 0.908, IFI = 0.909, and TLI = 0.895, which were judged appropriate [77].

**Figure 2:**
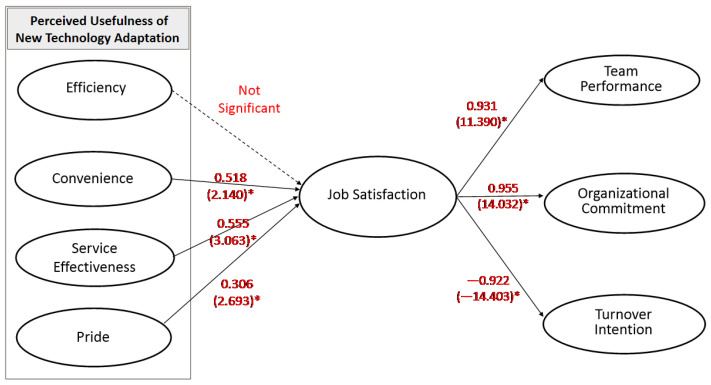
The Results of the Proposed Model (* *p* < 0.05).

Table 5 demonstrates the hypothesis testing results. All the hypotheses were supported, except H1. Figure 2 shows the results of the proposed model and the SEM with standardized theoretical path coefficient. According to the SEM, seven out of eight hypotheses were statically supported. Therefore, H1 is rejected (S.E. = 205, t = 0.910, *p* > 0.05). However, H2 (S.E. = 0.518, t = 2.140, *p* < 0.05), H3 (S.E. = 0.555, t = 3.063 *p* < 0.05), H4 (S.E. = 0.306, t = 2.693, *p* < 0.05), H5 (S.E. = 0.931, t = 11.390 *p* < 0.05), H6 (S.E. = 0.955, t = 14.032 *p* < 0.05), and H7 (S.E. = −0.922, t = −14.032 *p* < 0.05) are supported.

## 5. Discussion and Implications

Major airlines across the globe have been adopting new technologies and work platforms such as equipping flight attendants with a tablet PC, in order to provide diverse and differentiated services and enhance the travel experience of passengers. However, there have not yet been any academic findings and/or studies suggesting the enhancement of services and experiences with new technology adoption.

The CoIT suggests that organizations can benefit from the implementation of the BYOD concept and boost employee functionality at work [31]. This CoIT suggestion has been mainly supported by industries such as healthcare and educational services, but also by the airline industry and, specifically, by in-flight attendants.

This study reviews how the introduction of new technology and the use of tablet PCs by in-flight attendants can deliver potential cognitive effects/benefits to their job satisfaction. In addition, it illustrates how team performance, organizational commitment, and willingness to search for another job opportunity or turnover intention would be influenced by adopting these new technology and work platforms.

Based on the study, the potential cognitive effects/benefits are as follows: (1) efficiency, (2) convenience, and (3) pride. However, the adoption of new technology results in little or no significant impact on the simplification of flight preparation.

The theoretical implications of this study are as follows. Previous studies on the cognitive positive effects of the introduction of new technologies include a study in which the use of mobile devices by medical staff improved work efficiency in the healthcare field [32], a study in which the use of mobile devices by students in the educational field increased environmental sustainability [12], and research in which pride in the organization is shown as active service behavior [50]. However, as there are no studies on the aviation industry, this study is meaningful in that it is the first to theoretically reveal the cognitive positive effects of introducing new technologies to the work of flight attendants. Many airlines are also making financial investments to apply tablet PCs to flight attendants’ work, identifying which of the four types of cognitive positive effects plays a major role in bringing job satisfaction and providing academic value. In addition, combining IT and cognitive psychology will lay the foundation for the introduction of artificial intelligence technology in the future.

The practical l implications of this study are as follows. First, we were able to understand the overall global trends in which various airlines are increasingly adopting new technology for their flight attendants and considering how to meet and positively influence their job satisfaction. Accordingly, by combining ICT and aviation management, this study’s results have implications for more related research.

Second, the positive effect of the adaptation of new technology proven in this study can increase not only flight attendants’ competency but also customer satisfaction with airline services.

Third, the findings suggest strategies for airlines for flight attendants’ human resource management by reasoning about their job satisfaction through the adaptation of new technologies, and help improve flight attendants’ working conditions, corporate culture, and interpersonal relationships.

In conclusion, the results can provide and suggest practical implications for airlines that are currently considering whether to adopt new technology and leverage tablet PCs in the workplace. The COVID-19 pandemic has accelerated non-face-to-face services, and Koreans have preferred non-face-to-face travel to minimize the risk of COVID-19 infection [78]. In preparation for the post-COVID era, more airlines are creating a smart work environment with tablet PCs for passengers and flight attendants who prefer non-face-to-face services. This study provided a theoretical support for airlines worldwide to create a smart working environment in order to provide efficient services to passengers in the post-COVID-19 era.

## 6. Conclusions

We interpreted the results through the following aspects. First, the efficiency by adopting new technology does not positively influence job satisfaction. H1 is not a reasonable explanation of the potential cognitive effects/benefits from the adoption of new technology, because the actual data collection for flight preparation from various resources, including flight attendant manual books, is approximately 5 to 10 min on average. Therefore, it can be interpreted that the use of new technology such as a tablet PCs under the BYOD concept does not significantly simplify flight preparation or allow additional time prior to departure.

Second, the convenience of adopting new technology positively influences job satisfaction. The execution and task processing speed and transaction accuracy from the adoption of new technology are critical factors in job satisfaction. It is suggested that airlines should continuously monitor their technology to remain updated (e.g., processing speed of a tablet PC, battery life expectancy) and make additional investments in developing advanced mobile applications and software on their newly introduced work platforms.

Third, the service effectiveness generated by the adoption of new technology at work positively influences job satisfaction. Previous research shows that there is an enhanced work efficiency among medical staff who utilize a personal electronic device, as the utilization of those devices can deliver fast and effective communication and improve information retrieval [32,33,34,35,36,37,38,39]. For in-flight attendants, work efficiency is expected to be the most influential among other potential cognitive effects/benefits from the adoption of new technology. It is important to prioritize the efficiency of delivering exceptional onboard services by optimizing communication and service processes.

For example, the introduction of SkyTab tablets for in-flight attendants at Condor Airlines in 2020 has successfully optimized the airlines’ onboard sales process with cashless services using credit cards, Apple, and Google Pay. Through SkyTab’s in-flight sales technology, receipts can be digitally provided at Condor Airlines upon request. This new technology allows contactless services between in-flight attendants and their passengers while maintaining optimized communication with efficient logistics and onboard services.

Fourth, the pride of using new technology positively influences job satisfaction. Airlines should now consider how the introduction and adoption of a tablet PC can help employees take pride in their work while promoting higher job satisfaction. It was found that the intuitive part observed when cabin attendants use a tablet PC will elicit positive internal/external feedback to increase flight attendants’ pride and bring job satisfaction. For example, if the external design (color similar to corporate identity, size that is easy to carry, recognizable brand, etc.) is exceptional, customers may feel that they are an airline leading the latest IT technology, in line with the trend of global airlines, and that they are receiving a luxurious and meticulous service. Consequently, they will be satisfied with the service quality, and cabin attendants who have received positive internal/external feedback will experience pride, bringing greater job satisfaction.

This study has several limitations, based on which we suggest some directions for future research. First, the sample for this study consisted only of current in-flight attendants at one of the Korean airlines who have experience in using tablet PCs in the workplace. Therefore, these sampling limitations can affect the generalizability of this research study. To mitigate and overcome this limitation, there needs to be an increased sample size to include those at different airlines with tablet PC usage experiences and a comparable analysis study at each airline equipped with tablet PCs in the workplace.

Second, the sample for this study consisted of in-flight attendants and assistant pursers in the 30–39 age group only. Considering the difficulties and barriers of interacting with new technology among different age groups and ranks in the workplace, future researchers will need demographic information as a controlling factor for a more detailed study.

Third, COVID-19 was widespread at the time of the survey, which was conducted between August and September 2020. Therefore, it is possible that there may be a psychological effect on the survey responses of the flight attendants, owing to job insecurity and economic difficulties regarding an uncertain future. It would be preferable to reconsider the same survey after the aviation industry normalizes.

## Figures and Tables

**Table 1:** Characteristics of the Sample (N = 208).

Variables	Index	Frequency (n)	Percent (%)
Gender	Male	24	11.5
	Female	184	88.5
Age	20–29	41	19.7
	30–39	123	59.1
	40–49	39	18.8
	Over 50	5	2.4
Marital status	Single	90	43.3
	Married	118	56.7
Education	College degree	18	8.7
	University degree	158	76.0
	Graduate school	15	7.2
	Graduate degree	17	8.2
Working period	Less than 2 years	4	1.9
	2–5 years	25	12.0
	5–10 years	83	39.9
	10–15 years	49	23.6
	Over 15 years	47	22.6
Working grade	Attendant	90	43.3
	Assistant purser	84	40.4
	Purser	29	13.9
	Senior purser	3	1.4
	Chief purser	2	1.0
Average annual income	Less than KRW 30 million	5	2.4
	KRW 30–40 million	45	21.6
	KRW 40–50 million	83	39.9
	KRW 50–60 million	44	21.2
	Over KRW 60 million	31	14.9
Total		208	100.0

**Table 2:** Confirmatory Factor Analysis: Items and Loadings.

Construct and Scale Items		Factor Loading	Mean	Std dev.
Efficiency of flight preparation (3)	I think it is efficient to prepare the briefing before the flight at once with the use of tablet PC.	0.516	4.692	0.492
	With the use of tablet PC, information can be checked in advance of the flight, so I think we can provide customized services to our passengers.	0.603	4.519	0.589
	By using tablet PC, the time to prepare flight information is reduced, and there is more time to prepare flight-related tasks (apron, scarf, interview, etc.).	0.516	4.048	1.001
Convenience (3)	By using tablet PC, it is now possible to check information at a desired time compared to earlier.	0.616	1.408	0.582
	With the use of tablet PC, it is now possible to check information in a desired place compared to earlier.	0.619	1.375	0.616
	I think that the execution speed of tasks has been faster than before since using tablet PC.	0.780	1.548	0.672
	I think that the accuracy of tasks has been higher than before since using tablet PC.	0.769	1.596	0.688
Service effectiveness (3)	I think the quality of service has improved with the use of tablet PC.	0.803	4.038	0.821
	I think that the use of tablet PC has made it possible to communicate quickly with colleagues.	0.760	4.264	0.750
	I think that the use of tablet PC reduces the flow of services and is efficient.	0.809	4.057	0.991
Pride (4)	I feel proud of myself for using tablet PC in my work.	0.838	4.043	0.897
	I feel pride while using tablet PC when serving passengers.	0.907	4.014	0.975
	By using a tablet PC for work, I feel pride in comparison with other flight attendants in Korea.	0.880	4.101	0.950
	I feel pride that the company provided me with a tablet PC.	0.881	3.851	1.054
Job satisfaction (3)	The use of tablet PC makes work fun.	0.825	3.423	0.954
	I believe that the use of tablet PC helps personal growth.	0.850	3.461	1.025
	By using tablet PC, I am willing to recommend my company to my friends in the same field.	0.859	2.966	1.237
Team performance (3)	The use of tablet PC seems to have reduced our team’s minor mistakes in their work.	0.776	3.548	1.001
	The use of tablet PC seems to create a synergistic effect with the team members during team flight.	0.838	3.572	0.999
	The use of tablet PC seems to have a positive effect on overall team performance.	0.866	3.283	1.129
Organizational commitment (3)	Using tablet PC made me feel more connected to the company.	0.888	3.442	1.029
	Using tablet PC, I take pride in telling others about my company.	0.932	3.384	1.131
	Using tablet PC, I put in more effort than expected of myself for the company’s goal (improving service quality).	0.894	3.528	1.111
Turnover intention (3)	With the use of tablet PC, I intend to continue working at my current company.	0.885	2.644	1.132
	By using tablet PC, I think I can work for a longer duration because the burden of work is reduced.	0.920	2.543	1.132
	With the expectation that the functions of tablet PC will improve, I have a desire to continue working for the company.	0.838	2.355	1.057
χ^2^ = 677.930 (chi-square/df = 2.159, *p* < 0.001), CFI = 0.919, TLI = 0.903, RMSEA = 0.078				

Note: All factor loadings are significant at *p* < 0.001.

**Table 3:** Result of Convergent Validity.

Variations	C.R.	AVE
Efficiency	0.717	0.459
Convenience	0.905	0.708
Service effectiveness	0.874	0.698
Pride	0.935	0.782
Job satisfaction	0.850	0.654
Team performance	0.859	0.671
Organizational commitment	0.920	0.794
Turnover intention	0.896	0.743

Note: AVE: average variance extracted, C.R.: composite reliability.

**Table 4:** Descriptive Statistics and Associated Measures.

	AVE	1	2	3	4	5	6	7	8	9
Efficiency	0.459	0.717 ^a^								
Convenience	0.708	−0.830 ^b^ (0.689) ^c^	0.905							
Service effectiveness	0.698	0.703 (0.494)	−0.805 (0.648)	0.692 (0.479)	0.874					
Pride	0.782	0.68 (0.462)	−0.65 (0.423)	0.581 (0.338)	0.712 (0.507)	0.935				
Job satisfaction	0.654	0.596 (0.355)	−0.528 (0.279)	0.749 (0.561)	0.743 (0.552)	0.666 (0.444)	0.850			
Team performance	0.671	0.538 (0.289)	−0.578 (0.334)	0.776 (0.602)	0.764 (0.584)	0.703 (0.494)	0.893 (0.797)	0.859		
Organizational commitment	0.794	0.469 (0.220)	−0.441 (0.194)	0.644 (0.415)	0.705 (0.497)	0.724 (0.524)	0.897 (0.805)	0.892 (0.796)	0.920	
Turnover intention	0.743	−0.533 (0.284)	0.508 (0.258)	−0.567 (0.448)	−0.734 (0.539)	−0.669 (0.448)	−0.868 (0.753)	−0.839 (0.704)	−0.914 (0.835)	0.896

Note: AVE: average variance extracted. ^a^ Composite reliability is along the diagonal; ^b^ Correlations are above the diagonal; ^c^ Squared correlations are below the diagonal.

**Table 5:** Scandalized Parameter Estimates for Structural Model.

Paths			Hypothesis	β	S.E.	C.R.	*p*
Efficiency	→	Job satisfaction	Not supported	0.607	0.205	0.910	0.363
Convenience	→	Job satisfaction	Supported	1.092	0.518	2.140	0.032
Service effectiveness	→	Job satisfaction	Supported	0.634	0.555	3.063	0.002
Pride	→	Job satisfaction	Supported	0.306	0.306	2.693	0.007
Job satisfaction	→	Team performance	Supported	0.955	0.931	11.390	***
Job satisfaction	→	Organizational commitment	Supported	1.161	0.955	14.032	***
Job satisfaction	→	Turnover intention	Supported	−1.232	−0.922	−13.403	***

S.E.: Standardized estimate, C.R.: composite reliability. *** *p* < 0.001.

## Data Availability

No new data were created or analyzed in this study. Data sharing is not applicable to this article.
